# Internalized weight stigma, metabolic syndrome, and inflammation in postmenopausal women with obesity^☆^

**DOI:** 10.1016/j.bbih.2025.101129

**Published:** 2025-11-01

**Authors:** Rebecca L. Pearl, Stephen D. Anton, Danielle Saunders, Marian Hernandez, Laurie C. Groshon, Miriam Sheynblyum, Dakota L. Leget, Christian McLaren, Sarah Vial, Lecsy Gonzalez, Kevin Wu, Gayane Barsamyan, Thomas A. Wadden

**Affiliations:** a Department of Clinical and Health Psychology, College of Public Health and Health Professions, University of Florida, Gainesville, FL, USA; b Department of Physiology and Aging, College of Medicine, University of Florida, Gainesville, FL, USA; c Department of Medicine, College of Medicine, University of Florida, Gainesville, FL, USA; d Department of Psychiatry, Perelman School of Medicine, University of Pennsylvania, Philadelphia, PA, USA

**Keywords:** Health, Internalization, Self-stigma, Weight

## Abstract

**Objective:**

To determine the relationship of internalized weight stigma with metabolic syndrome (MetS) and markers of inflammation.

**Methods:**

Postmenopausal women with obesity (*N* = 101) with high or low scores on the Weight Bias Internalization Scale completed a single assessment visit. MetS components (waist circumference, blood pressure, triglycerides, HDL cholesterol, and glucose) were measured, along with body mass index (BMI). Blood samples were drawn to analyze C-reactive protein, interleukin-6, and myeloperoxidase. Participants completed a second measure of internalized weight stigma (Weight Self-Stigma Questionnaire; WSSQ) and reported medications, demographics, depression symptoms, smoking status, and perceived stress (included as covariates).

**Results:**

Logistic regression showed no significant relationship between either measure of internalized weight stigma and MetS when controlling for BMI, depression, and demographics. A small, significant, positive association emerged between WSSQ scores and MetS when adding the covariate of anti-depressant medication (OR = 1.07, *p* = 0.040). WSSQ scores were associated with significantly greater odds of having high blood pressure (OR = 1.10, *p* = 0.003) and low HDL cholesterol (OR = 1.06, *p* = 0.030). Both measures of internalized weight stigma were associated with greater continuous blood pressure values. Inflammatory markers were not associated with internalized weight stigma in most analyses.

**Conclusions:**

Some significant associations were found between internalized weight stigma and MetS components, but more research is needed to clarify these relationships.

## Highlights

• Internalized weight stigma was largely not associated with metabolic syndrome.
• Internalized weight stigma was associated with some metabolic syndrome components.
• In particular, internalized weight stigma was associated with high blood pressure.
• Internalized weight stigma was not associated with most inflammatory markers.

## 1 Introduction

Obesity is linked to heightened risk for cardiovascular disease, type 2 diabetes, and other metabolic and inflammatory diseases (Grundy, 2016). Many complex factors play a role in the relationship between obesity and poor health, including genetic, biological, physiological, psychological, social, and environmental factors (Siokou et al., 2014). One such factor that has gained increasing attention is weight stigma.

Weight stigma is rooted in negative attitudes, blame, and stereotypes against individuals with a high body weight or obesity and refers to the societal devaluation and mistreatment of individuals on the basis of their body weight (Pearl, 2018). Weight stigma occurs at the structural level – such as in societal norms or policies that promote or do not protect against unequal treatment due to weight – and at the interpersonal level – such as experiences of teasing, bullying, discrimination, or general mistreatment (Pearl, 2018). In addition to experiencing weight stigma from others, weight stigma also can be turned inward at the intrapersonal level, in a process known as *internalized weight stigma* (also called *weight bias internalization* or *weight self-stigma*; Durso and Latner, 2008; Lillis et al., 2010). Internalization occurs when individuals with obesity absorb derogatory societal messages about weight and apply these negative attitudes and stereotypes to themselves, leading to lower self-worth due to weight (Corrigan et al., 2006; Pearl and Puhl, 2018). Moderate to high levels of internalized weight stigma are reported by up to 50 % of U.S. adults with obesity (Puhl et al., 2018).

### 1.1 Weight stigma and obesity-related health

Weight stigma in all forms is strongly linked to poor mental health outcomes (Emmer et al., 2020). Much of the research on weight stigma and *physical health* outcomes has focused on *perceived discrimination*, or the subjective experience of being treated unfairly by others. Several studies have linked perceived weight discrimination with heightened risk of chronic diseases (e.g., arteriosclerosis; Daly et al., 2019; Udo et al., 2016) and with greater weight gain over time (Jackson et al., 2014; Sutin and Terracciano, 2013). For example, in a sample of 1365 adults with overweight and obesity, after controlling for demographics, body mass index (BMI), and other relevant health variables, perceived weight discrimination was associated with greater odds of having the *metabolic syndrome* (MetS*;* Adil et al., 2022). MetS is a cluster of risk factors which individually and together predict the onset of type 2 diabetes and cardiovascular disease (CVD; Cornier et al., 2008; Tune et al., 2017). Thus, weight stigma may be a risk factor for poorer cardiometabolic health among adults with overweight/obesity.

Compared to perceived discrimination, less is known about the effects of internalized weight stigma on physical health. Data from clinical and community samples have shown associations between internalized weight stigma and poor *self-rated* physical health (Pearl and Puhl, 2018). Few studies have investigated the relationship between internalized weight stigma and cardiometabolic health using objective, physiological measures. In one study that assessed a convenience sample of 159 treatment-seeking men and women with obesity, controlling for age, sex, race, BMI, and depression, participants who fell into the top tertile of levels of internalized weight stigma had three times greater odds of meeting criteria for MetS than those in the bottom tertile, and six times greater odds of having high triglycerides (Pearl et al., 2017). This remains the only study to investigate the relationship between internalized weight stigma and MetS and warrants replication.

### 1.2 Weight stigma, stress, and inflammation

Potential mechanisms that may explain the relationship between weight stigma and adverse health outcomes include availability of resources (which discrimination diminishes; Hatzenbuehler et al., 2013) and coping responses (such as eating; Himmelstein et al., 2018). A third proposed mechanism – stress – offers an overarching biobehavioral explanation for how chronic exposure to weight stigma, from external sources or from oneself, directly affects health. Since stigma is considered a psychosocial stressor (Busse et al., 2017; Mendes and Muscatell, 2018; Tomiyama, 2014), the effects of stress on the body are important to examine in relation to cardiometabolic health outcomes. Chronic activation of the hypothalamic-pituitary-adrenal (HPA) axis in response to stress contributes to the development of insulin resistance, inflammation, and impaired immune function, among other health impacts (Busse et al., 2017; Mendes and Muscatell, 2018; Tomiyama, 2014). In addition, chronic stress can result in oxidative stress (Betteridge, 2000), which leads to lipid accumulation and atherosclerotic plaque and causes CVD (Betteridge, 2000; Nicholls and Hazen, 2005). Indeed, perceived weight discrimination has been linked with heightened markers of inflammation and oxidative stress (Keirns et al., 2025; Sutin et al., 2014; Tomiyama et al., 2014). These findings support the Cyclic Obesity/Weight-Based Stigma (COBWEBS) Model, which describes how weight stigma-induced stress and its consequences (including physiological effects) contribute to weight gain and obesity (Tomiyama, 2014).

Individuals who self-stigmatize due to their weight may also have higher chronic stress due to constant self-criticism and expected rejection from others, even in the absence of discrete stigmatizing events (Brenchley and Quinn, 2016; Durso and Latner, 2008). However, few studies have investigated the relationship between internalized weight stigma and physiological stress. Two small studies of adults with obesity linked internalized weight stigma to dysregulated cortisol (Jung et al., 2020; Nicolau et al., 2023), with one study also finding an association with greater self-reported chronic stress (Jung et al., 2020); still, one of these studies found no association with other measures of inflammation (e.g., C-reactive protein; CRP; Nicolau et al., 2023). While the stress of experiencing stigma from others has been widely acknowledged and investigated (Lawrence et al., 2022), conceptualizing *self-directed stigma* as a form of chronic stress is novel and requires further examination.

### 1.3 Current study

The current study aimed to establish the cross-sectional relationship between internalized weight stigma and MetS and to explore potential stress-related mechanisms of this relationship. MetS is a widely used measure of CVD risk included as an outcome in studies of stress, inflammation, and obesity. Inflammation is a core mechanism in the development of MetS (Cornier et al., 2008). Specifically, the effects of interleukin-6 (IL-6) on glucose and lipid metabolism make it a key part of the pathogenetic pathway to MetS. CRP as a marker of chronic inflammation, and myeloperoxidase (MPO) as a marker of inflammation and oxidative stress, are also associated with components of MetS (Frohlich et al., 2000; Qaddoumi et al., 2020). Thus, IL-6, CRP, and MPO represent three stress-related biomarkers that are directly implicated in MetS and overall CVD risk. These markers have been associated with social stress (including perceived discrimination; Aschbacher et al., 2013; Cuevas et al., 2020; Sutin et al., 2014; Tomiyama et al., 2014), but no study to date has investigated whether internalized weight stigma is positively associated with IL-6, CRP, or MPO.

This study had two specific aims. The first aim was to replicate prior research showing an association between internalized weight stigma and MetS. The second aim was to determine the contribution of internalized weight stigma to heightened biomarkers of chronic stress and inflammation. To control for possible sex-related hormonal differences in inflammation and cardiometabolic risk factors (Carr, 2003; Thorand et al., 2006), this research was conducted in postmenopausal women due to greater prevalence and impacts of weight stigma in women compared to men (Himmelstein et al., 2017) and higher prevalence of MetS in middle to older adulthood compared to young adulthood (Hirode and Wong, 2020). We hypothesized that postmenopausal women with obesity who reported high (vs. low) levels of internalized weight stigma would have significantly greater odds of having MetS and would show heightened markers of inflammation.

## 2 Materials and methods

### 2.1 Participants

Participants were postmenopausal women with obesity recruited from the community to participate in a cross-sectional, observational study. Eligible participants had a BMI ≥30 kg/m^2^, were ages 45–65 years (older participants were excluded due to aging-related changes in inflammation; Sarkar and Fisher, 2006), and were postmenopausal women, defined as not experiencing a menstrual period for at least 12 consecutive months. In addition, participants were weight stable, defined as not having experienced a weight loss of ≥3 % of their initial weight in the last 3 months; with no history of bariatric surgery; and not taking medications that would affect weight such as prescription weight loss medications in the absence of type 2 diabetes, as well as steroids or nonsteroidal anti-inflammatory drugs, hormone replacement therapy medications, or certain neuropsychiatric medications due to their effects on inflammatory biomarkers. Participants also were not pregnant or nursing.

To replicate prior findings (Pearl et al., 2017), participants were selected to have either high or low levels of internalized weight stigma, as determined by the Weight Bias Internalization Scale (WBIS). Eligible participants had a score of 4.0 or greater (indicating elevated internalized weight stigma), or a score of 3.0 or lower (indicating low internalized weight stigma) on the WBIS. Those who scored between 3.0 and 4.0 on the WBIS were ineligible (see Appendix for more information).

### 2.2 Procedures

Participants were recruited through newspapers, flyers, social media, mailings, a community health center, and primary care clinics. Interested study candidates completed an online screening questionnaire through REDCap to assess BMI, internalized weight stigma, and other exclusion criteria. Following the online screening, potentially eligible candidates were screened by phone to confirm eligibility and, if eligible, scheduled to attend a single on-site assessment visit conducted in the morning with a study assessor masked to WBIS scores.

At the start of the visit, the study assessor confirmed that study candidates had fasted overnight for at least 8 h and measured height and weight in duplicate to confirm BMI. Weight was measured to the nearest 0.1 kg (with participants dressed in light clothing and without shoes) using a mechanical beam scale (Detecto). Height was measured to the nearest 0.1 cm. using a wall mounted stadiometer. If eligibility was confirmed, participants were consented. Following informed consent, the study assessor collected information by interview about medications, medical conditions, and current or recent infections. Waist circumference was measured in duplicate, as well as blood pressure and pulse, with a 5 min rest period before the blood pressure measurements and a 1 min rest period between measurements. Waist circumference was measured to the nearest 0.1 cm with a flexible tension-controlled measuring tape, positioned midway between the iliac crest and the lowest rib. Blood pressure and pulse were measured in duplicate using an automated Omron monitor at 1 min intervals after ≥5 min rest.

Questionnaires were administered online via REDCap. Blood draws were completed to obtain measures of fasting blood glucose, triglycerides, high-density lipoprotein (HDL) cholesterol, high sensitivity CRP, IL-6, and MPO. Participants were provided with a $50 pre-paid debit card, and lab results were emailed to participants. The study was approved by the institutional review board. Data collection occurred between December 2022 and May 2024.

### 2.3 Measures

#### 2.3.1 Internalized weight stigma

The 10-item Weight Bias Internalization Scale (WBIS) was used to categorize participants as having high vs. low internalized weigh stigma. The WBIS is the most widely used measure of internalized weight stigma (Pearl and Puhl, 2018) and assesses elements of internalization such as self-application of negative stereotypes and self-devaluation (Durso and Latner, 2008). The 10-item WBIS has strong psychometrics (Hilbert et al., 2014; Lee and Dedrick, 2016) and had high internal consistency in the current study (Cronbach's *α* = 0.92).

Based on WBIS scores, participants were divided into a “high” or “low” internalized weight stigma group (for more information, see Appendix). Dividing participants categorically into high vs. low internalized weight stigma is consistent with the previous study's suggestion of a threshold effect at which internalized weight stigma may correspond with heightened cardiometabolic disease risk (Pearl et al., 2017). This is also consistent with research that uses thresholds for diagnosis of syndromes and disorders (e.g., MetS, depression, etc.).

In addition to the WBIS, the Weight Self-Stigma Questionnaire (WSSQ) was included as a secondary, continuous measure of internalization. The WSSQ is also widely used and has strong psychometrics (Lillis et al., 2010). The scale consists of 12 items, rated 1–5, which are combined for a total score and also create two subscales: Fear of Enacted Stigma, which assesses anticipated and perceived stigma from others, and Self-Devaluation. In the current study, internal consistency of this measure and its subscales were acceptable to good (WSSQ Total score *α* = 0.89; Fear of Enacted Stigma subscale *α* = 0.90; Self-Devaluation subscale *α* = 0.77).

#### 2.3.2 MetS

The primary dependent variable was a diagnosis of MetS. The consensus definition of MetS from the International Diabetes Federation/American Heart Association/National Heart, Lung, and Blood Institute (Grundy, 2016) was used to determine whether participants met each individual criterion: elevated waist circumference (≥88 cm for women); high blood pressure (systolic ≥130 and/or diastolic ≥85 mmHg, and/or taking anti-hypertensive medication); elevated triglycerides (≥150 mg/dL and/or medication); reduced HDL cholesterol (<50 mg/dL for women and/or medication); and elevated fasting glucose (≥100 mg/dL and/or medication). Participants who met three of the five criteria were categorized as having MetS. Blood samples were collected as heparinized plasma and analyzed within 24 h.

#### 2.3.3 Inflammatory markers

Biomarkers of chronic stress and inflammation were high sensitivity CRP, IL-6, and MPO. CRP samples were collected as heparinized plasma and assayed within 24 h using latex agglutination on a Beckman Coulter AU5800 analyzer. IL-6 and MPO samples were collected as serum, stored at −80 °C, and analyzed using an enzyme-linked immunosorbent assay protocol (Quantikine HS600C and DMYEOOB).

#### 2.3.4 Covariates

Participants self-reported their age, race, ethnicity, and highest level of education. All of these variables are associated with both MetS and internalized weight stigma (Cornier et al., 2008; Pearl and Puhl, 2018). Based on the makeup of the sample, race was coded as White (vs. another race), ethnicity was coded as Hispanic/Latina (vs. not Hispanic/Latina), and education was coded as obtaining a bachelor's degree or higher (vs. not obtaining a bachelor's degree). BMI was also included in analyses to determine the contribution of internalized weight stigma to MetS and inflammation independent of the effects of adiposity.

Depression was included as a covariate due to associations with internalized weight stigma, MetS, and inflammation (Pan et al., 2012; Pearl and Puhl, 2018). The 8-item Patient Health Questionnaire-8 (PHQ-8; Kroenke et al., 2009) assessed the severity of symptoms of depression within the last two weeks on a scale of 0 (not at all) to 3 (nearly every day). Scores were summed, with higher scores indicating greater severity of depression (*α* = 0.80). Use of anti-depressant medication (reported by interview) was also included as a covariate in sensitivity analyses to account for depression and the medication's possible effects on inflammation. To account for general stress in analyses that examined inflammation, the 10-item Perceived Stress Scale (Cohen et al., 1983) assessed frequency of self-reported stress within the last month on a scale of 0 (never) to 4 (very often). Scores were summed, with higher scores indicating greater perceived stress (*α* = 0.61).

Participants reported by interview current or recent illnesses or infections, which were included as a covariate in analyses of inflammatory markers. Current medications were also reported; no medications that affect inflammation were reported beyond those captured in MetS criteria and anti-depressant medications. Smoking status (ever smoked vs. never smoked) was assessed for use in sensitivity analyses, due to the effects of smoking on inflammation.

### 2.4 Statistical analyses

Sample and power calculations were based on prior data of the association between the primary outcome variable MetS (yes/no) and the main independent variable of internalized weight stigma (high/low). In the only prior study on this topic (Pearl et al., 2017), participants with high stigma (compared to low) had an odds ratio (OR) of 3.2 for meeting MetS criteria when controlling for BMI, depression, age, sex, and race/ethnicity (approximately 40 % of participants with high stigma and 20 % with low stigma met criteria for MetS, unadjusted OR = 2.7). Using the standard Wald-type test for logistic regression outlined by Demidenko (2007), a power analysis was conducted using GPower 3.1. Based on these data, we conservatively estimated an OR of 2.7, with the expectation (based on prior data) that covariates would account for 0.25 of the variance in WBIS scores. A sample size of 107 participants was determined to be needed to provide 90 % power to detect a significant between-group difference with a two-tailed alpha = 0.05. Our recruitment goal was 110 participants (55 with high and low internalized weight stigma, respectively). Secondary outcomes were considered exploratory.

Data quality and integrity were checked by assessing data for missing and out-of-range values. Continuous measures were checked for violations in the normality assumption. If violations were detected, variance-stabilizing transformations were used, and sensitivity analyses removed statistical outliers using the 1.5 interquartile range method to improve normality (see Appendix). Analyses were conducted for participants who had complete data for the variables needed in those specific analyses. Preliminary analyses compared participant demographic characteristics, BMI, and depression by whether participants had high vs. low WBIS scores (using t-tests for continuous variables and Chi-Square test for categorical data). Regardless of imbalances, these variables were included as covariates in the final analyses.

The primary analysis was conducted using logistic regression to determine the effect of internalized weight stigma on the diagnosis of MetS. Regression models added covariates in blocks (BMI and depression, and age, race, ethnicity, and education). Supplemental analyses used the same regression models to examine the effects of internalized weight stigma on each of the five MetS criteria (in five separate models), and post-hoc analyses used linear regression to consider effects on continuous measures of MetS criteria. Exploratory analyses replaced the independent predictor of high vs. low internalized weight stigma (based on WBIS scores) with the WSSQ total score as a secondary, continuous measure of internalized weight stigma, along with the Self-Devaluation and Fear of Enacted Stigma subscales. Sensitivity analyses also controlled for potential confounds of anti-depressant medication and smoking status.

Linear regression was used for the secondary aim of testing the relationship between internalized weight stigma and CRP, IL-6, and MPO, respectively (in three separate models). Covariates were added in steps (BMI and depression, demographics, and recent illnesses/infections). Exploratory and sensitivity analyses that were conducted for the primary aim were repeated with each inflammatory marker as the dependent variable. Exploratory analyses also controlled for perceived stress to determine if the association between internalized stigma and inflammation persists beyond general stress. Analyses were two-tailed with a significance level of 0.05 and were conducted with SPSS version 30. Data are available upon reasonable request.

## 3 Results

Table 1 presents a summary of participant characteristics (see Appendix, Figure A.1 for recruitment flow chart). Due to unanticipated challenges recruiting participants with low internalized weight stigma, a final sample of 101 participants was obtained: 55 participants were categorized as having a high level of internalized weight stigma, and 46 participants were categorized as having low internalized weight stigma. Significant differences were found between participants with high vs. low WBIS scores for race, BMI, and depression. Specifically, participants in the category of having high WBIS scores were more likely to be White vs. another race (χ^2^ = 5.56, *p* = 0.018) and had significantly higher BMI (39.75±7.78 kg/m^2^ vs. 36.85±4.49 kg/m^2^, *p* = 0.028) and PHQ-8 scores (7.82±4.19 vs. 4.15±4.05, *p* < 0.001). Age, ethnicity, and education were not significantly associated with having a high vs. low WBIS score. WBIS scores and WSSQ scores were strongly correlated (*r* = 0.60, 0.60, and 0.44, *p* < 0.001 for WSSQ total score and Fear of Enacted Stigma and Self-Devaluation subscales, respectively).

**Table 1:** Participant characteristics, by categories of high vs. low scores on the Weight Bias Internalization Scale (WBIS).

Variables	Mean ± SD or N (%)		
	High WBIS (*N* = 55)	Low WBIS (*N* = 46)	Total Sample (*N* = 101)
Age (years)	57.38±4.82	58.35±4.22	57.82 ± 4.56
Body Mass Index (kg/m^2^)	39.75±7.78	36.85±4.49	38.43 ± 6.62
Race			
White	46 (83.6 %)	29 (63.0 %)	75 (74.3 %)
Black or African American	6 (10.9 %)	13 (28.3 %)	19 (18.8 %)
American Indian or Alaska Native	1 (1.8 %)	1 (2.2 %)	2 (2.0 %)
Asian	0	1 (2.2 %)	1 (1.0 %)
More than one race	2 (3.6 %)	1 (2.2 %)	3 (3.0 %)
Unknown	0	1 (2.2 %)	1 (1.0 %)
Hispanic or Latina	7 (12.7 %)	5 (10.9 %)	12 (11.9 %)
Highest Level of Education			
Some high school	1 (1.8 %)	1 (2.2 %)	2 (2.0 %)
High school diploma or GED	3 (5.5 %)	3 (6.5 %)	6 (5.9 %)
Some college or technical/trade/vocational school (no degree)	11 (20.0 %)	12 (26.1 %)	23 (22.8 %)
Associate's degree	7 (12.7 %)	5 (10.9 %)	12 (11.9 %)
Bachelor's degree	15 (27.3 %)	15 (32.6 %)	30 (29.7 %)
Some graduate school (no degree)	1 (1.8 %)	1 (2.2 %)	2 (2.0 %)
Master's degree	12 (21.8 %)	5 (10.9 %)	17 (16.8 %)
Professional, doctoral, or other advanced degree	5 (9.1 %)	4 (8.7 %)	9 (8.9 %)
Patient Health Questionnaire-8	7.82±4.19	4.15±4.05	6.15±4.50
Use of anti-depressant medication	17 (30.9 %)	9 (19.6 %)	26 (25.7 %)
Reported a current or recent illness or infection	4 (7.3 %)	3 (6.5 %)	7 (6.9 %)
Smoking Status			
Never smoked	33 (60.0 %)	29 (63.0 %)	62 (61.4 %)
Formerly smoked	20 (36.4 %)	14 (30.4 %)	34 (33.7 %)
Currently smokes	2 (3.6 %)	3 (6.5 %)	5 (5.0 %)
Weight Bias Internalization Scale	4.87±0.74	2.35±0.56	–
Weight Self-Stigma Questionnaire	35.22±9.32	24.83±8.04	30.49±10.15
Self-Devaluation Subscale	16.69±4.47	12.91±4.44	14.97±4.82
Fear of Enacted Stigma Subscale	18.53±6.39	11.91±5.00	15.52±6.65
Participants meeting criteria for metabolic syndrome:			
Waist Circumference	55 (100.0 %)	45 (97.8 %)	100 (99.0 %)
Blood Pressure	39 (70.9 %)	32 (69.6 %)	71 (70.3 %)
Triglycerides	35 (63.6 %)	24 (52.2 %)	59 (58.4 %)
HDL Cholesterol	34 (61.8 %)	26 (56.5 %)	60 (59.4 %)
Glucose	24 (43.6 %)	27 (58.7 %)	51 (50.5 %)
Diagnosis of metabolic syndrome (3 or more criteria)	41 (74.5 %)	32 (69.6 %)	73 (72.3 %)
C-Reactive Protein	6.96±5.49	6.65±7.09	6.82±6.23
Interleukin-6	2.85±1.27	3.84±3.98	3.30±2.87
Myeloperoxidase	127.72±95.03	143.60±114.23	134.95±103.98

Note. High WBIS was defined as a score of 4 or above on the 1–7 scale. Low WBIS was defined as a score of 3 or below. For C-reactive protein, *N* = 100 due to removal of one outlier in the low WBIS group (*N* = 45).

### 3.1 Internalized weight stigma and MetS

In the primary logistic regression model, high (vs. low) WBIS scores were not significantly associated with MetS in any step of the model (when covariates were included, OR = 1.38, 95 % CI = 0.49–3.90, *p* = 0.54). No covariate was associated with MetS. Sensitivity analyses controlling for smoking status or anti-depressant medication did not change the results.

Exploratory analyses that replaced categories of high vs. low WBIS scores with the total score on the WSSQ also did not yield significant results (with covariates, OR = 1.06, 95 % CI = 0.997–1.123, *p* = 0.064). Results were not significant for the WSSQ Fear of Enacted Stigma subscale (OR = 1.08, 95 % CI = 0.99–1.17, *p* = 0.09) or the WSSQ Self-Devaluation subscale (OR = 1.08, 95 % CI = 0.96–1.21, *p* = 0.19). Controlling for smoking status did not change the results. However, when adding use of anti-depressant medication as a covariate, the effect of WSSQ total scores on the diagnosis of MetS became significant, with higher WSSQ scores associated with 7 % greater odds of meeting criteria for MetS: OR = 1.07, 95 % CI = 1.003–1.137, *p* = 0.040 (Table A.1). Results for the WSSQ subscales remained non-significant.

Overall, high WBIS scores were not associated with MetS, and WSSQ total scores showed a small, positive significant relationship with MetS only when controlling for all covariates, including use of anti-depressant medication.

#### 3.1.1 Individual MetS criteria

Five logistic regression models tested associations between high (vs. low) WBIS scores and whether participants met each criterion for MetS. Results were not significant for waist circumference, blood pressure, triglycerides, or HDL cholesterol. High WBIS scores were associated with lower odds of having high glucose when controlling for BMI and depression (OR = 0.33, 95 % CI = 0.13–0.86, *p* = 0.022), but this effect was no longer significant when demographic characteristics were included in the model (OR = 0.40, 95 % CI = 0.15 = 1.07, *p* = 0.066). Controlling for smoking or anti-depressant use did not change the results.

Analyses were repeated replacing the WBIS categories with the WSSQ total score and its two subscales. WSSQ total scores were significantly associated with 10 % greater odds of having high blood pressure (*p* = 0.003; Table 2). Effects were significant for both the Self-Devaluation (*p* = 0.017) and Fear of Enacted Stigma subscales (*p* = 0.021). In addition, WSSQ total scores were significantly associated with 6 % greater odds of having low HDL cholesterol when controlling for all covariates (*p* = 0.03; Table 3). Results were not significant for the Self-Devaluation subscale (*p* = 0.053) or the Fear of Enacted Stigma subscale (*p* = 0.074). Results were not significant for waist circumference, triglycerides, or glucose. Results did not change when controlling for smoking status or anti-depressant medication use.

**Table 2:** Logistic regression models of associations between Weight Self-Stigma Questionnaire (WSSQ) scores and odds of having high blood pressure or taking anti-hypertensive medication.

Step	Variable	Odds Ratio	95 % CI	p value	Nagelkerke R-Square
Model 1					
1	**WSSQ Total**	**1.07**	**1.02–1.12**	**0.009**	0.10
2	**WSSQ Total**	**1.06**	**1.01–1.12**	**0.034**	0.17
	**BMI**	**49.91**	**1.10–2264.93**	**0.045**	
	PHQ-8	0.97	0.86–1.09	0.57	
3	**WSSQ Total**	**1.10**	**1.03–1.18**	**0.003**	0.29
	BMI	14.54	0.29–718.86	0.18	
	PHQ-8	0.95	0.84–1.08	0.43	
	Age	1.06	0.96–1.18	0.25	
	**Bachelor's Degree**	**0.32**	**0.11–0.96**	**0.042**	
	White	0.36	0.11–1.25	0.11	
	Hispanic/Latina	0.62	0.14–2.68	0.52	
Model 2					
1	**WSSQ-SD**	**1.13**	**1.02–1.24**	**0.017**	0.09
2	**WSSQ-SD**	**1.13**	**1.01–1.27**	**0.029**	0.18
	**BMI**	**90.62**	**2.08–3950.34**	**0.019**	
	PHQ-8	0.97	0.86–1.09	0.60	
3	**WSSQ-SD**	**1.22**	**1.07–1.40**	**0.004**	0.29
	BMI	40.68	0.82–2030.18	0.06	
	PHQ-8	0.95	0.84–1.08	0.43	
	Age	1.08	0.97–1.20	0.15	
	Bachelor's Degree	0.41	0.14–1.20	0.10	
	White	0.34	0.09–1.20	0.09	
	Hispanic/Latina	0.63	0.14–2.83	0.55	
Model 3					
1	**WSSQ-FE**	**1.09**	**1.01–1.16**	**0.021**	0.08
2	WSSQ-FE	1.07	0.98–1.15	0.12	0.14
	**BMI**	**52.80**	**1.16–2404.64**	**0.042**	
	PHQ-8	0.99	0.89–1.11	0.88	
3	**WSSQ-FE**	**1.11**	**1.02–1.22**	**0.020**	0.24
	BMI	14.12	0.28–708.06	0.19	
	PHQ-8	0.99	0.89–1.11	0.89	
	Age	1.05	0.95–1.16	0.33	
	Bachelor's Degree	0.34	0.12–1.02	0.054	
	White	0.50	0.16–1.57	0.24	
	Hispanic/Latina	0.61	0.15–2.47	0.48	

Note. BMI values were transformed with the natural log. WSSQ=Weight Self-Stigma Questionnaire; SD=Self-Devaluation subscale; FE= Fear of Enacted Stigma subscale; BMI=Body Mass Index; PHQ-8 = Patient Health Questionnaire-8.

**Table 3:** Logistic regression models of associations between Weight Self-Stigma Questionnaire (WSSQ) scores and odds of having low HDL cholesterol or taking medication to manage cholesterol.

Step	Variable	Odds Ratio	95 % CI	p value	Nagelkerke R-Square
Model 1					
1	WSSQ Total	1.02	0.98–1.07	0.24	0.02
2	WSSQ Total	1.04	0.99–1.01	0.09	0.05
	BMI	0.25	0.02–3.75	0.32	
	PHQ-8	0.94	0.85–1.05	0.28	
3	**WSSQ Total**	**1.06**	**1.01–1.13**	**0.030**	0.13
	BMI	0.13	0.01–2.89	0.20	
	PHQ-8	0.93	0.83–1.04	0.21	
	Age	0.97	0.88–1.07	0.53	
	Bachelor's Degree	1.18	0.47–2.95	0.72	
	**White**	**0.28**	**0.10–0.85**	**0.024**	
	Hispanic/Latina	0.46	0.11–1.92	0.28	
Model 2					
1	WSSQ-SD	1.05	0.96–1.14	0.28	0.02
2	WSSQ-SD	1.07	0.98–1.18	0.15	0.04
	BMI	0.39	0.03–5.23	0.48	
	PHQ-8	0.95	0.86–1.06	0.36	
3	WSSQ-SD	1.11	0.998–1.238	0.053	0.12
	BMI	0.29	0.02–5.12	0.40	
	PHQ-8	0.94	0.84–1.05	0.27	
	Age	0.98	0.89–1.01	0.66	
	Bachelor's Degree	1.37	0.56–3.36	0.49	
	**White**	**0.29**	**0.10–0.87**	**0.027**	
	Hispanic/Latina	0.47	0.11–1.91	0.29	
Model 3					
1	WSSQ-FE	1.03	0.97–1.10	0.31	0.01
2	WSSQ-FE	1.06	0.98–1.14	0.13	0.04
	BMI	0.24	0.02–3.80	0.31	
	PHQ-8	0.96	0.87–1.06	0.39	
3	WSSQ-FE	1.07	0.99–1.16	0.07	0.11
	BMI	0.13	0.01–3.16	0.21	
	PHQ-8	0.95	0.86–1.06	0.36	
	Age	0.97	0.88–1.06	0.46	
	Bachelor's Degree	1.19	0.48–2.99	0.71	
	**White**	**0.34**	**0.12–0.96**	**0.041**	
	Hispanic/Latina	0.45	0.11–1.82	0.26	

Note. BMI values were transformed with the natural log. WSSQ=Weight Self-Stigma Questionnaire; SD=Self-Devaluation subscale; FE= Fear of Enacted Stigma subscale; BMI=Body Mass Index; PHQ-8 = Patient Health Questionnaire-8.

In sum, high WBIS scores were not associated with individual MetS criteria when controlling for covariates. WSSQ scores were significantly associated with having high blood pressure and low HDL cholesterol when controlling for covariates.

#### 3.1.2 Post-hoc analyses

Linear regression models included the continuous values for all individual MetS criteria (and controlled for medication use relevant to each criterion). Results showed that participants in the category of high (vs. low) WBIS scores had significantly higher levels of systolic blood pressure (β = 0.24, *p* = 0.046; Table A.2). High WBIS scores were also associated with higher waist circumference values when BMI was not included in the model (due to its strong correlation with waist circumference; β = 0.25, *p* = 0.022). Triglycerides were significantly associated with high WBIS scores when controlling for medications to treat dyslipidemia (β = 0.21, *p* = 0.038), but these effects were not significant when other covariates were included in the models. High WBIS scores were not associated with continuous measures of HDL cholesterol or glucose. Results did not change when controlling for smoking status or use of anti-depressant medication.

Similarly, higher WSSQ total scores were associated with significantly higher levels of diastolic blood pressure (β = 0.29, *p* = 0.028; Table A.3). This effect was driven by the Self-Devaluation subscale (β = 0.35, *p* = 0.004) but was not significant for the Fear of Enacted stigma subscale (*p* = 0.28). WSSQ total scores and Fear of Enacted Stigma subscale scores were significantly associated with higher waist circumference values when BMI was not included in the models (*β* = 0.24 and 0.28, *p* = 0.035 and 0.008, respectively). WSSQ total scores were significantly associated with higher triglycerides when controlling for dyslipidemia medication (β = 0.22, *p* = 0.031), but not when controlling for the other covariates. WSSQ scores were not associated with HDL cholesterol, or with glucose when using a transformed variable due to non-normality. However, when statistical outliers were removed instead, glucose levels were significantly associated with higher WSSQ Self-Devaluation scores (β = 0.25, *p* = 0.039). This effect was no longer significant when use of anti-depressant medication was included in the model (no other effects were changed when including this covariate or smoking status).

Linear regression models also tested whether the total number of MetS criteria met by participants (out of the five possible criteria) differed based on participants’ level of internalized weight stigma. Results were not significant when participants were categorized based on high vs. low WBIS scores (*p* = 0.93). However, WSSQ total scores were associated with meeting significantly more MetS criteria (controlling for all covariates, β = 0.29, *p* = 0.023; Table A.4). Results were significant for the WSSQ Self-Devaluation subscale (β = 0.25, *p* = 0.023) but not for the WSSQ Fear of Enacted Stigma subscale (*p* = 0.077), although the latter became significant when also controlling for use of anti-depressant medication (β = 0.26, *p* = 0.043). Otherwise, results were consistent when controlling for smoking status or anti-depressant medication use.

In sum, both high WBIS scores and WSSQ scores were associated with higher continuous levels of blood pressure and waist circumference. Continuous measures of triglycerides, HDL cholesterol, and glucose were not associated with either internalized weight stigma measure when controlling for all covariates. WSSQ scores (but not high WBIS scores) were positively associated with meeting criteria for a greater number of MetS components, controlling for all covariates.

### 3.2 Internalized weight stigma and inflammation

WBIS and WSSQ scores were not significantly associated with CRP in any analysis. Similarly, neither measure was associated with IL-6. WBIS and WSSQ scores were not significantly associated with MPO when values were transformed to improve normality. In sensitivity analyses that instead removed statistical outliers to improve normality, WSSQ total scores and Self-Devaluation subscale scores were significantly associated with higher levels of MPO (β = 0.27 and 0.26, *p* = 0.036 and 0.031, respectively; Table A.5). Results did not change for any analysis of inflammatory markers when controlling for anti-depressant medication use, smoking status, or perceived stress. Overall, internalized weight stigma was not associated with inflammation in most analyses.

## 4 Discussion

The current study aimed to determine the relationship of internalized weight stigma with MetS, and to explore its potential links to biomarkers of stress and inflammation that could explain such a relationship. This research was based on prior findings of a strong association between high levels of internalized weight stigma and the diagnosis of MetS – as well as high triglycerides – in a clinical, convenience sample of adults with obesity (Pearl et al., 2017). In the current community sample of postmenopausal women with obesity, these previous findings largely did not replicate. Based on our primary measure of internalized weight stigma (the WBIS), no significant differences were observed in the diagnosis of MetS between participants with high vs. low levels of internalized weight stigma. Only in additional analyses in which a secondary measure of internalized weight stigma was used (the WSSQ) and all covariates were added did a significant relationship emerge. The strength of this relationship was smaller than in the prior study, with higher WSSQ scores in the current study associated with 7 % higher odds of having MetS, compared to a three-fold increase in odds in the prior study (Pearl et al., 2017).

A positive association between internalized weight stigma and high blood pressure was the most consistent finding in the current study, emerging as significant in most (though not all) analyses. WSSQ scores (but not WBIS scores) were associated with significantly greater odds of having high blood pressure and low HDL cholesterol. In post-hoc analyses, both the WBIS and the WSSQ showed significant, positive associations with continuous levels of blood pressure. For the WSSQ, this effect appeared to be driven by the Self-Devaluation subscale. This finding is consistent with prior research linking stress related to experiencing weight stigma from others to higher blood pressure (Major et al., 2012; Panza et al., 2023). The possibility that self-directed weight stigma may carry similar associations with high blood pressure as experiencing weight stigma from external sources is a novel finding and warrants further examination in future research.

In post-hoc analyses, both measures of internalized weight stigma were also significantly associated with higher waist circumference when BMI was not included in the models. A prior study of 70 adults ages 18–50 years similarly found greater visceral adipose tissue among women with higher levels of internalized weight stigma (Keirns et al., 2022). Criticisms of BMI as an insufficient measure to assess health risks have led to calls for the use of other metrics, including waist circumference (Sweatt et al., 2024). Waist circumference, as a measure of central adiposity, is a stronger predictor of poor health outcomes than BMI (Sweatt et al., 2024). Our findings thus support the link between internalized weight stigma and risk for cardiometabolic disease. Almost all participants in the current sample met the categorical criterion for having an elevated waist circumference, which likely explained the lack of significant differences by internalized weight stigma in categorical analyses. Still, the significant relationship between continuous values of waist circumference and internalized weight stigma, all among adults with obesity, is notable and suggests links between these two factors.

Other MetS criteria such as glucose, triglycerides, and HDL cholesterol were significantly associated with internalized weight stigma in some analyses, but these effects were not as robust. The total number of MetS criteria met was significantly associated with higher WSSQ scores, but not with categorization of high vs. low internalized weight stigma based on WBIS scores. Overall, given the inconsistency of these findings, no firm conclusions can be drawn about the relationship between internalized weight stigma and these other health indicators. Prior research on experiencing weight discrimination has also found non-significant associations with specific components of MetS (Adil et al., 2022), emphasizing the need for more research to clarify the associations between weight stigma and MetS.

Finally, the current study did not find significant associations between internalized weight stigma and biomarkers of stress and inflammation in most analyses – with the exception of a positive association between MPO and WSSQ scores when outliers were removed. One prior study also did not find a significant relationship between WBIS scores and markers of inflammation (CRP and ferritin; Nicolau et al., 2023). Although consistent links are observed overall between perceived discrimination and stress-related inflammatory markers, associations with CRP and IL-6 have been somewhat mixed (Kershaw et al., 2016; Lawrence et al., 2022). Given the limited work in this area, more research is needed to understand possible biological pathways by which internalized weight stigma may impact health.

The current study had several strengths, including its use of objective measures of health and inflammation, a tightly controlled design that aimed to eliminate possible confounds (e.g., related to sex or age), and use of multiple measures of internalized weight stigma and several sensitivity analyses to determine robustness of results. Still, there were a number of limitations. Due to challenges recruiting individuals with low levels of internalized stigma, we did not reach our intended sample size and may have been underpowered to detect significant effects. The observed effects were also smaller than expected based on prior research, so bigger samples may be needed to determine if small but significant effects are present. Larger population-based studies that include measures of internalized weight stigma may be needed to replicate findings with sufficient power. Discrepancies in findings with the WBIS versus the WSSQ were notable and were consistent with previously observed differences in associations between these measures and weight-related health outcomes (Pearl, 2024). The WBIS and WSSQ measure somewhat different aspects of internalized weight stigma, which may have accounted for different results: The WBIS assesses negative self-directed attitudes, including those related to appearance; the WSSQ assesses anticipated and perceived stigma (in the Fear of Enacted Stigma subscale) and self-devaluation. Furthermore, WSSQ scores were treated continuously in this study (vs. the high and low WBIS categories based on cut-off scores), meaning that there was greater statistical power to detect small effects.

The focus on postmenopausal women in the current study served to control for sex differences and hormonal factors that could affect inflammation and cardiometabolic health, but results cannot be generalized to other populations. The current study also did not exclude adults with type 2 diabetes, since that would have limited the range of possible glucose values; the prior study on this topic did exclude individuals with type 2 diabetes (Pearl et al., 2017), which may account in part for the divergent results (along with other differences between the two samples). This study was also observational and cross-sectional, so directionality and causality cannot be inferred. Prospective cohort studies and intervention studies that test the effects of reducing internalized weight stigma on cardiometabolic health outcomes are needed to elucidate temporality and causality in the relationships between internalized weight stigma, MetS, and markers of stress and inflammation.

### 4.1 Conclusion

The current study of postmenopausal women with obesity did not demonstrate a robust relationship between internalized weight stigma and MetS. Some analyses did show a relationship between internalized weight stigma and specific components of MetS, especially a novel association with high blood pressure that has not been shown in previous studies. Combined with prior findings, these results support the need to further explore this relationship in larger samples and prospective or experimental studies. Given previously established links between internalized weight stigma and poor weight-related health outcomes, testing and dissemination of interventions to reduce internalized weight stigma is recommended (Pearl, 2024). Clinical screening and public health efforts to address obesity may benefit from greater consideration of internalized weight stigma as a facet of obesity-related health.

## CRediT authorship contribution statement

**Rebecca L. Pearl:** Writing – original draft, Visualization, Supervision, Project administration, Methodology, Investigation, Funding acquisition, Formal analysis, Data curation, Conceptualization. **Stephen D. Anton:** Writing – review & editing, Resources, Methodology, Conceptualization. **Danielle Saunders:** Writing – original draft, Validation, Project administration, Investigation. **Marian Hernandez:** Writing – review & editing, Validation, Software, Project administration, Investigation. **Laurie C. Groshon:** Writing – review & editing, Investigation. **Miriam Sheynblyum:** Writing – review & editing, Investigation. **Dakota L. Leget:** Writing – review & editing, Investigation. **Christian McLaren:** Writing – review & editing, Investigation. **Sarah Vial:** Writing – review & editing, Investigation. **Lecsy Gonzalez:** Writing – review & editing, Investigation. **Kevin Wu:** Writing – review & editing, Investigation. **Gayane Barsamyan:** Writing – review & editing, Investigation. **Thomas A. Wadden:** Writing – review & editing, Methodology, Conceptualization.

## Funding

This research was supported by the National Heart, Lung, And Blood Institute of the National Institutes of Health under Award R03HL160603. The content is solely the responsibility of the authors and does not necessarily represent the official views of the National Institutes of Health.

## Declaration of competing interest

The authors declare the following financial interests/personal relationships which may be considered as potential competing interests: Thomas Wadden previously served on a scientific advisory board for Weight Watchers and receives grant support, on behalf of the University of Pennsylvania, from Novo Nordisk. If there are other authors, they declare that they have no known competing financial interests or personal relationships that could have appeared to influence the work reported in this paper.

## Data Availability

Data will be made available on request.
