# Supplementary material for: Internalized weight stigma, metabolic syndrome, and inflammation in postmenopausal women with obesity
Source: Brain Behav Immun Health. 2025 Nov 1;50:101129. doi: 10.1016/j.bbih.2025.101129 (PMC12637082; doi:10.1016/j.bbih.2025.101129)
Supplement: Multimedia component 1 [file mmc1.docx]

**Appendix A: Supplementary Material**

**Pearl RL, Anton SD, Saunders D, Hernandez M, Groshon LC, Sheynblyum M, Leget DL, McLaren C, Vial S, Gonzalez L, Wu K, Barsamyan G, Wadden TA (2025). Internalized Weight Stigma, Metabolic Syndrome, and Inflammation in Postmenopausal Women with Obesity. Brain, Behavior, and Immunity – Health.**

**Additional Details about Study Methods**

***Weight Bias Internalization Scale (WBIS).*** The high stigma cutoff score of 4.0 (the midpoint of the WBIS) has been used in prior studies of individuals with high internalization (Pearl et al., 2020, 2023). This score indicates that, on average, participants do not disagree with self-stigmatizing statements. Based on population norms, a score of 4.0 represents the 80^th^ percentile of scores for women with overweight/obesity (Hilbert et al., 2014). It also approximates the cutoff for the top tertile of WBIS scores among participants in the only prior study of the relationship between internalized weight stigma and MetS (Pearl et al., 2017). A score of 3.0 was used as the upper limit for categorizing participants with “low” internalized weight stigma. Population norm data suggest that this represents approximately the 55^th^ percentile for women (Hilbert et al., 2014), providing separation from the high stigma cutoff. This score also corresponds with the cutoff of the bottom tertile of WBIS scores in the previous MetS study (Pearl et al., 2017).

***Anthropometric and Clinical Measurements.*** If the first two measurements of waist circumference differed by more than 0.5 cm, a third measurement was taken, and the two measurements that were closest were averaged. Similarly, a third blood pressure measurement was obtained if ≥160/90 or if there was a significant difference in two readings of SBP ≥15 or DBP ≥10, averaging the two closest measurements.

***Blood Collection and Processing.*** Blood was collected via venipuncture. Samples for glucose, cholesterol, triglycerides, and hs-CRP were collected in a 3mL Lithium heparin tube and immediately transported to a laboratory for analysis. One 5mL SST tube was used to collect samples for IL-6 and MPO. The SST tube was allowed to clot, then centrifuged (Eppendorf 5810R) and separated into three aliquots, which were transported within 1-2 weeks for storage at -80°C for future analysis.

**References:**

Hilbert, A., Baldofski, S., Zenger, M., Lowe, B., Kersting, A., & Braehler, E. (2014). Weight Bias Internalization Scale: Psychometric properties and population norms. *PLos ONE*, *9*(1), e86303.

Pearl, R. L., Wadden, T. A., Bach, C., Gruber, K., Leonard, S., Walsh, O. A., Tronieri, J. S., & Berkowitz, R. I. (2020). Effects of a cognitive-behavioral intervention targeting weight stigma: A randomized controlled trial. *Journal of Consulting and Clinical Psychology*, *88*(5), 470–480.

Pearl, R. L., Wadden, T. A., Bach, C., LaFata, E. M., Gautam, S., Leonard, S., Berkowitz, R. I., Latner, J. D., & Jakicic, J. M. (2023). Long-term effects of an internalized weight stigma intervention: A randomized controlled trial. *Journal of Consulting and Clinical Psychology*, *91*(7), 398-410.

Pearl, R. L., Wadden, T. A., Hopkins, C. M., Shaw, J. A., Hayes, M. R., Bakizada, Z. M., Alfaris, N., Chao, A. M., Pinkasavage, E., Berkowitz, R. I., & Alamuddin, N. (2017). Association between weight bias internalization and metabolic syndrome among treatment-seeking individuals with obesity. *Obesity*, *25*(2), 317–322.

**Additional Details about Data Inspection and Transformation**

BMI was skewed and transformed with the natural log. Triglycerides, glucose, IL-6, and MPO values were also skewed, due in part to several outliers that all had plausible values. One CRP value (144) was removed as an outlier due to the possibility that it reflected a lab error or severe infection but was still not normally distributed. These variables were all transformed with the natural log; in sensitivity analyses, instead of transformation, statistical outliers were removed using the 1.5 interquartile range method, which improved normality for all variables.

Figure A.1. CONSORT participant flow chart

5,819 participants entered the pre-screening survey

5,330 excluded based on inclusion/exclusion criteria

1,329 incomplete or blank survey

1,007 BMI<30

883 had not been through menopause

655 taking hormone replacement therapy

483 high WBIS score after filling quota

276 WBIS score between 3 and 4

237 taking anti-inflammatory medication

213 taking weight loss medications/supplements

189 underwent bariatric surgery

24 outside of age range

21 pregnant/nursing

12 did not identify as a female

1 enrolled in weight loss treatment

489 participants eligible for phone screening

158 did not complete the phone screening

132 lost to follow up

26 no longer interested

331 participants completed the phone screening

168 excluded based on inclusion/exclusion criteria

80 taking exclusionary medication or recent change in medication

32 recent weight loss of 3% or more

23 unable to attend study visit

11 taking hormone replacement therapy

9 enrolled in weight loss treatment

8 BMI<30

3 had not been through menopause

2 undergoing chemotherapy or radiation

163 participants eligible after phone screening

14 lost to follow up

149 participants scheduled the assessment visit

42 excluded before completing assessment visit

36 lost to follow up

6 taking exclusionary medication

107 participants completed the assessment visit

3 excluded due to taking exclusionary medications

3 excluded due to error in WBIS scoring

101 participants included in the analyses

55 High WBIS Score

46 Low WBIS Score

Table A.1. Sensitivity analysis: Logistic regression model of association between Weight Self-Stigma Questionnaire (WSSQ) scores and the presence of metabolic syndrome, controlling for use of anti-depressant medication

| Step | Variable | Odds Ratio | 95% CI | p value | Nagelkerke R-Square |
| --- | --- | --- | --- | --- | --- |
| 1 | WSSQ Total | 1.04 | 0.99-1.08 | 0.14 | 0.03 |
| 2 | WSSQ Total | 1.05 | 0.99-1.11 | 0.09 | 0.06 |
|  | BMI | 0.17 | 0.01-3.54 | 0.25 |  |
|  | PHQ-8 | 0.99 | 0.87-1.11 | 0.81 |  |
|  | Anti-Depressant | 1.75 | 0.56-5.50 | 0.34 |  |
| 3 | **WSSQ Total** | **1.07** | **1.003-1.137** | **0.040** | 0.12 |
|  | BMI | 0.07 | 0.002-2.28 | 0.14 |  |
|  | PHQ-8 | 0.97 | 0.85-1.11 | 0.66 |  |
|  | Anti-Depressant | 1.89 | 0.59-6.12 | 0.29 |  |
|  | Age | 0.95 | 0.85-1.05 | 0.32 |  |
|  | Bachelor’s Degree | 1.01 | 0.38-2.72 | 0.98 |  |
|  | White | 0.37 | 0.11-1.25 | 0.11 |  |
|  | Hispanic/Latina | 0.47 | 0.10-2.16 | 0.33 |  |

Note. BMI values were transformed with the natural log. WSSQ=Weight Self-Stigma Questionnaire; BMI=Body Mass Index; PHQ-8=Patient Health Questionnaire-8

Table A.2. Post-hoc linear regression analyses examining relationship between high (vs. low) Weight Bias Internalization Scale (WBIS) scores and continuous values of metabolic syndrome components

| Variables | Waist Circumference | | Systolic Blood Pressure | | Diastolic Blood Pressure | | Triglycerides | | HDL Cholesterol | | Glucose | |
| --- | --- | --- | --- | --- | --- | --- | --- | --- | --- | --- | --- | --- |
|  | Beta | p | Beta | p | Beta | p | Beta | p | Beta | p | Beta | p |
| High WBIS | **0.25** | **0.022** | **0.24** | **0.046** | 0.17 | 0.16 | 0.12 | 0.28 | -0.01 | 0.91 | -0.08 | 0.43 |
| Medication | -- | -- | 0.12 | 0.26 | 0.16 | 0.16 | -0.02 | 0.86 | -0.04 | 0.70 | **0.38** | **<0.001** |
| BMI | -- | -- | -0.03 | 0.79 | -0.05 | 0.64 | -0.04 | 0.69 | -0.11 | 0.32 | -0.06 | 0.57 |
| PHQ-8 | 0.13 | 0.21 | -0.04 | 0.70 | 0.02 | 0.85 | 0.09 | 0.41 | 0.02 | 0.84 | **0.22** | **0.036** |
| Age | -0.06 | 0.50 | 0.17 | 0.10 | -0.10 | 0.34 | 0.01 | 0.91 | 0.07 | 0.53 | **-0.23** | **0.015** |
| Bachelor’s Degree | -0.16 | 0.10 | -0.04 | 0.71 | 0.01 | 0.92 | 0.09 | 0.43 | -0.01 | 0.93 | -0.08 | 0.43 |
| White | -0.01 | 0.94 | -0.08 | 0.48 | -0.03 | 0.82 | **0.22** | **0.038** | 0.06 | 0.60 | -0.03 | 0.76 |
| Hispanic/Latina | **-0.27** | **0.006** | -0.02 | 0.87 | -0.11 | 0.32 | 0.08 | 0.44 | -0.12 | 0.29 | 0.09 | 0.34 |

Note. Relevant medications were controlled for in each model (e.g., anti-hypertensive medication was controlled for in the models testing associations between WBIS scores and blood pressure). BMI, triglycerides, and glucose values were transformed with the natural log. Additional analyses (not shown) removed statistical outliers for the latter two variables instead of transformation to improve normality.

Table A.3. Post-hoc linear regression analyses examining relationship between Weight Self-Stigma Questionnaire (WSSQ) total scores and continuous values of metabolic syndrome

| Variables | Waist Circumference | | Systolic Blood Pressure | | Diastolic Blood Pressure | | Triglycerides | | HDL Cholesterol | | Glucose | |
| --- | --- | --- | --- | --- | --- | --- | --- | --- | --- | --- | --- | --- |
|  | Beta | p | Beta | p | Beta | p | Beta | p | Beta | p | Beta | p |
| WSSQ Total | **0.24** | **0.035** | 0.13 | 0.34 | **0.29** | **0.028** | 0.12 | 0.33 | -0.23 | 0.08 | -0.01 | 0.95 |
| Medication | -- | -- | 0.07 | 0.54 | 0.09 | 0.42 | -0.02 | 0.89 | -0.04 | 0.75 | **0.39** | **<0.001** |
| BMI | -- | -- | -0.01 | 0.91 | -0.09 | 0.44 | -0.06 | 0.62 | -0.06 | 0.63 | -0.07 | 0.50 |
| PHQ-8 | 0.12 | 0.29 | -0.01 | 0.94 | -0.04 | 0.73 | 0.09 | 0.46 | 0.12 | 0.32 | 0.19 | 0.08 |
| Age | -0.08 | 0.42 | 0.16 | 0.13 | -0.10 | 0.32 | 0.003 | 0.97 | 0.07 | 0.53 | **-0.22** | **0.018** |
| Bachelor’s Degree | **-0.20** | **0.046** | -0.07 | 0.56 | -0.05 | 0.66 | 0.06 | 0.58 | 0.03 | 0.77 | -0.08 | 0.44 |
| White | -0.002 | 0.98 | -0.06 | 0.61 | -0.06 | 0.58 | **0.23** | **0.035** | 0.11 | 0.32 | -0.05 | 0.64 |
| Hispanic/Latina | **-0.26** | **0.010** | -0.02 | 0.87 | -0.11 | 0.32 | 0.09 | 0.42 | -0.13 | 0.26 | 0.09 | 0.35 |

Note. Relevant medications were controlled for in each model (e.g., anti-hypertensive medication was controlled for in the models testing associations between WBIS scores and blood pressure). BMI, triglycerides, and glucose values were transformed with the natural log. Additional analyses (not shown) removed statistical outliers for these two variables to create a more normal distribution.

Table A.4. Post-hoc linear regression analysis examining associations between Weight Self-Stigma Questionnaire (WSSQ) scores and total number of metabolic syndrome criteria met by participants

| Step | Variable | Beta | p value | R-Square | Adjusted R-Square |
| --- | --- | --- | --- | --- | --- |
| Model 1 | | | | | |
| 1 | **WSSQ Total** | **0.22** | **0.030** | 0.05 | 0.04 |
| 2 | WSSQ Total | 0.21 | 0.07 | 0.05 | 0.03 |
|  | BMI | 0.004 | 0.97 |  |  |
|  | PHQ-8 | 0.01 | 0.96 |  |  |
| 3 | **WSSQ Total** | **0.29** | **0.023** | 0.10 | 0.03 |
|  | BMI | -0.02 | 0.89 |  |  |
|  | PHQ-8 | -0.03 | 0.83 |  |  |
|  | Age | 0.05 | 0.66 |  |  |
|  | Bachelor’s Degree | 0.02 | 0.89 |  |  |
|  | **White** | **-0.24** | **0.025** |  |  |
|  | Hispanic/Latina | -0.05 | 0.64 |  |  |
| Model 2 | | | | | |
| 1 | WSSQ-SD | 0.19 | 0.053 | 0.04 | 0.03 |
| 2 | WSSQ-SD | 0.18 | 0.12 | 0.04 | 0.01 |
|  | BMI | 0.04 | 0.72 |  |  |
|  | PHQ-8 | 0.02 | 0.84 |  |  |
| 3 | **WSSQ-SD** | **0.25** | **0.034** | 0.09 | 0.02 |
|  | BMI | 0.04 | 0.72 |  |  |
|  | PHQ-8 | -0.01 | 0.92 |  |  |
|  | Age | 0.06 | 0.55 |  |  |
|  | Bachelor’s Degree | 0.05 | 0.62 |  |  |
|  | **White** | **-0.24** | **0.026** |  |  |
|  | Hispanic/Latina | -0.05 | 0.65 |  |  |
| Model 3 | | | | | |
| 1 | WSSQ-FE | 0.019 | 0.057 | 0.04 | 0.03 |
| 2 | WSSQ-FE | 0.017 | 0.13 | 0.04 | 0.01 |
|  | BMI | 0.003 | 0.98 |  |  |
|  | PHQ-8 | 0.038 | 0.73 |  |  |
| 3 | WSSQ-FE | 0.22 | 0.08 | 0.08 | 0.01 |
|  | BMI | -0.01 | 0.91 |  |  |
|  | PHQ-8 | 0.03 | 0.82 |  |  |
|  | Age | 0.03 | 0.74 |  |  |
|  | Bachelor’s Degree | 0.02 | 0.85 |  |  |
|  | **White** | **-0.21** | **0.049** |  |  |
|  | Hispanic/Latina | -0.05 | 0.62 |  |  |

Note. BMI values were transformed with the natural log. WSSQ=Weight Self-Stigma Questionnaire; SD=Self-Devaluation subscale; FE= Fear of Enacted Stigma subscale; BMI=Body Mass Index; PHQ-8=Patient Health Questionnaire-8

Table A.5. Linear regression model testing the association between Weight Self-Stigma Questionnaire total scores and myeloperoxidase when statistical outliers were removed

| Step | Variable | Beta | p value | R-Square | Adjusted R-Square |
| --- | --- | --- | --- | --- | --- |
| 1 | WSSQ Total | 0.20 | 0.055 | 0.04 | 0.03 |
| 2 | **WSSQ Total** | **0.27** | **0.023** | 0.14 | 0.11 |
|  | **BMI** | **0.23** | **0.026** |  |  |
|  | **PHQ-8** | **-0.25** | **0.029** |  |  |
| 3 | **WSSQ Total** | **0.27** | **0.037** | 0.17 | 0.10 |
|  | **BMI** | **0.27** | **0.016** |  |  |
|  | **PHQ-8** | **-0.28** | **0.022** |  |  |
|  | Age | 0.01 | 0.95 |  |  |
|  | Bachelor’s Degree | 0.15 | 0.15 |  |  |
|  | White | -0.13 | 0.23 |  |  |
|  | Hispanic/Latina | 0.003 | 0.98 |  |  |
| 4 | **WSSQ Total** | **0.27** | **0.036** | 0.18 | 0.10 |
|  | **BMI** | **0.26** | **0.021** |  |  |
|  | **PHQ-8** | **-0.27** | **0.028** |  |  |
|  | Age | 0.01 | 0.94 |  |  |
|  | Bachelor’s Degree | 0.16 | 0.15 |  |  |
|  | White | -0.13 | 0.21 |  |  |
|  | Hispanic/Latina | 0.01 | 0.96 |  |  |
|  | Illnesses or Infections | 0.08 | 0.42 |  |  |
